# Regression analysis of gait parameters and mobility measures in a healthy cohort for subject-specific normative values

**DOI:** 10.1371/journal.pone.0199215

**Published:** 2018-06-18

**Authors:** Val Mikos, Shih-Cheng Yen, Arthur Tay, Chun-Huat Heng, Chloe Lau Ha Chung, Sylvia Hui Xin Liew, Dawn May Leng Tan, Wing Lok Au

**Affiliations:** 1 Department of Electrical and Computer Engineering, National University of Singapore, Singapore, Singapore; 2 Department of Physiotherapy, Tan Tock Seng Hospital, Singapore, Singapore; 3 Department of Physiotherapy, Singapore General Hospital, Singapore, Singapore; 4 Department of Neurology, National Neuroscience Institute, Singapore, Singapore; The Ohio State University, UNITED STATES

## Abstract

**Background:**

Deviation in gait performance from normative data of healthy cohorts is used to quantify gait ability. However, normative data is influenced by anthropometry and such differences among subjects impede accurate assessment. De-correlation of anthropometry from gait parameters and mobility measures is therefore desirable.

**Methods:**

87 (42 male) healthy subjects varying form 21 to 84 years of age were assessed on gait parameters (cadence, ankle velocity, stride time, stride length) and mobility measures (the 3-meter/7-meter Timed Up-and-Go, 10-meter Walk Test). Multiple linear regression models were derived for each gait parameter and mobility measure, with anthropometric measurements (age, height, body mass, gender) and self-selected walking speed as independent variables. The resulting models were used to normalize the gait parameters and mobility measures. The normalization’s capability in de-correlating data and reducing data dispersion were evaluated.

**Results:**

Gait parameters were predominantly influenced by height and walking speed, while mobility measures were affected by age and walking speed. Normalization de-correlated data from anthropometric measurements from |*r*_*s*_| < 0.74 to |*r*_*s*_| < 0.23, and reduced data dispersion by up to 69%.

**Conclusion:**

Normalization of gait parameters and mobility measures through linear regression models augment the capability to compare subjects with varying anthropometric measurements.

## Introduction

Physiotherapists commonly rely on mobility measures for a patient’s assessment, such as the 3-meter/7-meter Timed Up-and-Go (3mTUG/7mTUG) [1] and the 10-meter Walk Test (10mWT) [2]. These mobility measures have been employed to enable goal-setting [3], to assess gait capacity [4], to evaluate rehabilitation progresses [5, 6], or to gauge a Parkinson’s disease patient’s falls risk [7]. Patients’ performance on these mobility measures is used to assess the severity of their pathological gait by quantifying the degree to which their performance deviates from normative data of an age-matched healthy cohort [8]. Hence, accurate normative data is crucial for proper physiotherapeutic assessment and characterization of gait.

With regard to the South-East Asian population, knowledge about normative gait data is scarce and fragmented. Although age and gender-specific normative values for TUG and gait speed have been widely reported, these studies largely involved healthy cohorts residing in the United States [2, 9–13] or Europe [14–17]. Gait ability, however, may be influenced by ethnicity or country due to differences in anthropometric measurements. Indeed, normative values for the 3mTUG of Japanese cohorts were 1.44 s faster on average than those of Caucasian cohorts in a summary of 14 studies [18]. Hence, physiotherapeutic assessment of gait parameters and mobility measures is impeded for South-East Asian subjects as they rely on normative data of non-South-East Asian cohorts with different anthropometric measurements.

A possible remedy is to de-correlate anthropometric measurements from the data through normalization, thereby eliminating any influence of anthropometric differences among patients. Various methods have been proposed for normalization: Hof et al. demonstrated how gait data can be normalized to a non-dimensional quantity by accounting for an individual’s height and weight [19]. In contrast, the detrending technique proposed by O’Malley et al. preserves the data’s units while successfully eliminating any correlation between anthropometric measurements and gait data [20]. A more sophisticated approach attempts to normalize the data through the use of multiple regression models, which was shown by Wahid et al. to surpass the aforementioned methods in the ability to de-correlate anthropometric measurements from gait data [21].

In this study, we derive multiple linear regression models for various gait parameters and mobility measures of a healthy Singaporean cohort. The regression models are then used to normalize the data according to Wahid et al.’s method [21]. The effect of anthropometric measurements on gait parameters and mobility measures is evaluated, and the ability of the normalization process to de-correlate the data and reduce the dispersion within data is demonstrated. Anthropometric measurements evaluated are age, height, body mass and gender. We also included the subject’s self-selected walking speed as an independent variable, as it was shown to significantly affect gait parameters [21–23]. The demonstrated normalization has the potential to improve gait related analyses, such as the differentiation of Parkinsonian gait from healthy controls [21], or the evaluation of spatio-temporal parameters of cohorts comprising children of varying ages [24, 25].

## Methods

### Subjects

87 healthy subjects participated in the study for the acquisition of gait parameters and mobility measures. All subjects were Singaporean and recruited via convenience sampling between May 2011 and September 2012. The inclusion criteria were adults aged 21 years and above, without central or peripheral nervous system disorders, and no significant orthopaedic or rheumatological disorders affecting walking ability. Subjects were excluded if they were unable to provide informed consent, were pregnant, had implanted devices or had sensitive skin conditions that prohibited them from using the gait monitoring system. The subject demographics are displayed in Table 1. A goodness-of-fit test using the Kolmogorov–Smirnov statistic (*D*) for the continuous variables (age, height, body mass, walking speed) and Chi-Squared statistic (*χ*^2^) for the categorical variable (gender) indicates the uniform distribution of age and gender. Each subject had his unique self-selected walking speed derived from the average velocity observed in all 10mWT trials. Four additional subjects were recruited for the sole purpose of validating the gait monitoring system. Two of them were Parkinson’s disease patients in anticipation of future studies involving subjects with Parkinson’s disease. The study was approved by the Singhealth Centralized Institutional Review Board (CIRB 2011/255/A) and all subjects gave written informed consent.

**Table 1:** Anthropometry measurements and demographic parameters of the 87 recruited subjects along with its corresponding distribution.

Demographics	Data Ranges		Distribution		
	*μ* ± *σ*	Range	Fit	*D* or *χ*^2^	*α*
Age (years)	49.2 ± 16.8	[21, 83]	uniform	0.13	0.10
Height (cm)	164 ± 9	[148, 189]	normal	0.11	0.20
Body Mass (kg)	63.2 ± 15.1	[45, 117]	normal	0.14	0.05
Walking Speed (m/s)	1.34 ± 0.22	[0.83,1.80]	normal	0.05	0.20
Gender	42 male, 45 female		uniform	0.10	0.05

### Study procedure

Subjects performed the 3mTUG and 7mTUG thrice each, and 10mWT twice at their comfortable pace on a firm and flat surface at the Motor Control Laboratory. The TUG was performed with an arm chair, and a turning point marked at 3-meter from the chair (for the 3mTUG) and another at 7-meter from the chair (for the 7mTUG). The 7mTUG was included because longer walking distances are recommended for Parkinson’s disease assessments and hence normative values thereof become essential [26]. Subjects were instructed to sit with their back leaning against the back rest, and to stand up and walk upon the command ‘Ready, Go’. They were allowed to use the arm rest if they could not rise from the chair without support. The time to complete the task was manually recorded by a physiotherapist using a stopwatch, from the ‘Go’ signal to the time when the subject returned to the chair and sat down with the back leaning against the back rest. The 10mWT was performed with subjects walking a total distance of 14 meters, with start-time at the 2-meter mark and end-time at the 12-meter mark in order to remove acceleration and deceleration phases.

### Extraction of gait parameters and mobility measures

All subjects performed the TUG and 10mWT while wearing a wireless gait monitoring system [27]. In brief, the system consists of three sensor nodes, one of which is placed around the neck using a neck holder while the other two are placed around the ankles using a flexible strap. Each sensor node consists of a microcontroller (AVR ATMega328), 3-axis accelerometer (BMA180), 3-axis gyroscope (ITG3200), 3-axis digital compass (HMC5843) and a wireless module for data transmission to a data-logging device. Acceleration, angular velocity and local magnetic field are captured with a sampling rate of 100 Hz and transmitted using Bluetooth with a baud rate of 57600 bps. A sensor node weighs roughly 15 g and transmits data to the data-logging device which can be located 15 to 25 meters away. Mobility measures extracted with the gait monitoring system included time taken (in seconds) to complete the 3mTUG, 7mTUG and 10mWT, as well as the sit-to-stand and stand-to-sit duration. The extracted gait parameters included cadence (steps/min), ankle velocity (deg/s), stride time (s) and stride length (m). Gait parameters were extracted from a patient’s 10mWT, whereas sit-to-stand and stand-to-sit durations were extracted from the TUG tests. The gait parameters and mobility measures were evaluated for every successfully completed trial and no average among multiple trials were calculated.

### Statistics

The gait monitoring system is examined for its test-retest reliability and validated through criterion validation using intra-class correlation coefficients (ICC). The test-retest reliability is computed for every gait parameter and mobility measure among a subject’s trials with ICC(3,1) [28]. To evaluate the system’s criterion validity, the agreement between the gait monitoring system and a gold standard is computed using ICC(2,1). For gait parameters, the gold standard is a 3D motion analysis system (Qualysis Motion Analysis System, Gothenburg, Sweden), whereas for mobility measures, the gold standard is given by a therapist’s measurements. Reliability and agreement are deemed satisfactory if the corresponding ICC value exceeds 0.75 [29].

The multiple linear regression model selection followed a backward elimination process. First, variance inflation factors (VIF) were computed to check for potential multicollinearity. If the VIF exceeded 5, the independent variables were screened for correlations and removed from the model. The elimination process then removes the least significant independent variable from the model, given that it is not significant at p < 0.001. This process is repeated until only significant variables remain, or the adjusted *R*^2^ drops below 95% of the *R*^2^ observed from the model comprising all independent variables. To check whether the resulting models are overfitted, a 10-fold cross-validated root-mean-square error (CV-RMSE) is computed and compared to the resulting model’s RMSE. Given the resulting multiple linear regression model, the normalization of gait parameters and mobility measures is obtained by [21]

$$\begin{array}{c} y_{norm}=\frac{y_{raw}}{y_{model}}, \end{array}$$ (1)

where *y*_*raw*_ is the raw data value and *y*_*model*_ is the subject-specific predicted data value using the resulting multiple linear regression. Finally, the statistical assumptions of a linear regression, namely linearity, homoscedasticity and normality have been met for each independent variable.

The linear regression model’s ability to de-correlate gait parameters and mobility measures from anthropometric measurements through normalization is evaluated using Spearman correlation coefficients before and after normalization. To further evaluate the normalization’s ability to reduce dispersion within the data, the coefficient of variation is computed along with its 95% confidence interval (CI) and standard error (SE). Differences between pre- and post-normalization outcomes were evaluated using the student’s t-test with p < 0.05. All analyses were performed using MATLAB (version R2014a).

## Results

### Criterion validity and test-retest reliability of the gait monitoring system

Table 2 shows the cohort’s mean values of the extracted gait parameters and mobility measures, as well as their agreement with the gold standard (ICC(2,1)) and their test-retest reliability (ICC(3,1)). In general, the test-retest reliability of the various measures was good, with ICC(3,1) > 0.75 except for stand-to-sit duration (ICC(3,1) = 0.67). The agreement of the gait monitoring system with the gold standard was excellent, with no ICC(2,1) dropping below 0.96 for gait parameters and mobility measures. No agreement to a therapist’s measurement was evaluated for sit-to-stand or stand-to-sit, as manual measurement of these measures proved difficult. The analysis for ankle velocity was omitted here, as angular measurements were validated in our previous study [30].

**Table 2:** The criterion validity (agreement to 3D motion analysis system/a physiotherapist’s measurements, ICC(2,1)) and test-retest reliability among a subject’s trials (ICC(3,1)).

Gait & Mobility Measures	*μ* ± *σ*	ICC(2,1)	ICC(3,1)
***Mobility Measures***			
3mTUG (s)	9.61 ± 1.68	0.94	0.82
7mTUG (s)	14.88 ± 2.05	0.97	0.94
10mWT (s)	7.68 ± 1.25	0.96	0.82
sit-to-stand (s)	1.18 ± 0.33	-	0.76
stand-to-sit (s)	2.08 ± 0.52	-	0.67
***Gait Parameters***			
cadence (steps/min)	120.22 ± 10.75	0.99	0.91
ankle velocity (deg/s)	217.61 ± 20.98	–	–
stride time (s)	1.01 ± 0.09	0.97	0.88
stride length (m)	1.31 ± 0.12	0.96	0.75

### Multiple linear regression models

The resulting multiple linear regression models are displayed in Table 3. A subject’s self-selected walking speed remained significant for every gait parameter and mobility measure. Gait parameters were furthermore affected by a subject’s height, while mobility measures were influenced predominantly by a subject’s age. Gender was only significant for ankle velocity and sit-to-stand, but both models are performing rather poor compared to others (adjusted *R*^2^ ≤ 0.270). Removal of the gender variable in these models reduces the adjusted *R*^2^ significantly, hence the backward elimination process retained the model containing the gender variable. With exception of stand-to-sit, all other models demonstrate a good ability to predict the gait parameters and mobility measures. The CV-RMSE are only slightly higher than the model’s RMSE, indicating that the linear regression models are not overfitted. As walking speed correlates with age (*r*_*s*_ = -0.38, p < 0.05), linear regression models without walking speed have been added to allow for a more accurate discussion on the impact of age on gait parameters and mobility measures. However, the use of walking speed is advisable for de-correlation of data and reduction in data dispersion through normalization as the resulting models are more potent.

**Table 3:** Resulting multiple linear regression models for the mobility measures and gait parameters. The adjusted *R*^2^ is shown along with the root-mean square error (RMSE) and the 10-fold cross-validated RMSE (CV-RMSE). All models and remaining independent variables are significant at p < 0.001. The variables selected are walking speed (*S*), height (*H*), age (*A*) and gender (*G*).

Dependent Variable	Multiple Linear Regression Model					Adjusted *R*^2^	RMSE	CV-RMSE
***Mobility Measures (with walking speed (S))***								
3mTUG (s)	= 8.501	− 4.418 ⋅ *S*	+ 0.034 ⋅ *H*	+ 0.029 ⋅ *A*		0.473	1.205	1.221
7mTUG (s)	= 17.321	− 6.344 ⋅ *S*	+ 0.022 ⋅ *H*	+ 0.039 ⋅ *A*		0.532	1.496	1.497
10mWT (s)	= 15.537	− 5.902 ⋅ *S*				0.904	0.378	0.388
sit-to-stand (s)	= 1.262	− 0.371 ⋅ *S*		+ 0.006 ⋅ *A*	+ 0.160 ⋅ *G*	0.226	0.292	0.293
stand-to-sit (s)	= 2.391	− 0.471 ⋅ *S*		+ 0.006 ⋅ *A*		0.084	0.499	0.506
***Gait Parameters (with walking speed (S))***								
cadence (steps/min)	= 159.200	+ 39.833 ⋅ *S*	− 0.567 ⋅ *H*			0.763	5.240	5.335
ankle velocity (deg/s)	= 286.250	+ 47.675 ⋅ *S*	− 0.736 ⋅ *H*		− 20.633 ⋅ *G*	0.270	17.921	18.282
stride time (s)	= 0.660	− 0.345 ⋅ *S*	+ 0.005 ⋅ *H*			0.735	0.049	0.050
stride length (m)	= −0.238	+ 0.418 ⋅ *S*	+ 0.005 ⋅ *H*			0.667	0.068	0.069
***Mobility Measures (without walking speed (S))***								
3mTUG (s)	= −1.248		+ 0.051 ⋅ *H*	+ 0.049 ⋅ *A*		0.252	1.436	1.444
7mTUG (s)	= 3.228		+ 0.048 ⋅ *H*	+ 0.070 ⋅ *A*		0.276	1.868	1.870
10mWT (s)	= 6.378			+ 0.028 ⋅ *A*		0.142	1.128	1.134
sit-to-stand (s)	= 0.678			+ 0.008 ⋅ *A*	+ 0.146 ⋅ *G*	0.193	0.298	0.301
stand-to-sit (s)	= 1.637			+ 0.009 ⋅ *A*		0.066	0.504	0.511
***Gait Parameters (without walking speed (S))***								
cadence (steps/min)	= 244.310		− 0.669 ⋅ *H*	− 0.306 ⋅ *A*		0.398	8.342	8.402
ankle velocity (deg/s)	= 324.990		− 0.598 ⋅ *H*		− 17.100 ⋅ *G*	0.060	20.264	20.524
stride time (s)	= −0.088		+ 0.006 ⋅ *H*	+ 0.003 ⋅ *A*		0.402	0.073	0.075
stride length (m)	= 0.197		+ 0.006 ⋅ *H*			0.181	0.106	0.107

### De-correlation through normalization

The correlations of anthropometric measurements with gait parameters and mobility measures are shown in Table 4. The mobility measures had significant correlations with age and walking speed before normalization. A subject’s body mass also significantly correlated with the TUG tests and the sit-to-stand motion. After normalization, no significant correlation between anthropometric measurements or walking speed and mobility measures remained. The correlation coefficients *r*_*s*_ reduced from |*r*_*s*_| < 0.45 to |*r*_*s*_| < 0.06 for age, and even |*r*_*s*_| < 0.97 to |*r*_*s*_| < 0.07 for walking speed. Overall, the correlations for mobility measures dropped from |*r*_*s*_| < 0.97 to |*r*_*s*_| < 0.14 after normalization. Similarly, normalization removed any significant correlation between anthropometric measurements or walking speed and gait parameters, with the exception of age that remained significant for cadence and stride time. Nonetheless, significant correlations involving height, body mass, walking speed and gender have all been successfully removed. Overall, the correlations for gait parameters dropped from |*r*_*s*_| < 0.74 to |*r*_*s*_| < 0.23 after normalization.

**Table 4:** Spearman correlation coefficients for the data before (raw) and after normalization. Significance level p < 0.05.

Correlations	Age		Height		Body Mass		Walking Speed		Gender	
	Raw	Norm	Raw	Norm	Raw	Norm	Raw	Norm	Raw	Norm
***Mobility Measures***										
3mTUG (s)	0.45	-0.01^†^	0.10^†^	-0.01^†^	0.14	-0.07^†^	-0.63	-0.07^†^	-0.11^†^	0.07^†^
7mTUG (s)	0.44	-0.06^†^	0.03^†^	0.00^†^	0.19	0.05^†^	-0.67	0.06^†^	-0.07^†^	0.08^†^
10mWT (s)	0.35	0.04^†^	0.06^†^	-0.12^†^	0.14^†^	-0.12^†^	-0.97	0.04^†^	-0.12^†^	0.14^†^
sit-to-stand (s)	0.35	-0.04^†^	-0.21	0.02^†^	-0.25	-0.11^†^	-0.33	-0.02^†^	0.19	-0.02^†^
stand-to-sit (s)	0.26	0.00^†^	0.01^†^	0.06^†^	0.01^†^	0.00^†^	-0.26	-0.01^†^	0.03^†^	0.04^†^
***Gait Parameters***										
cadence (steps/min)	-0.35	-0.23	-0.42	-0.03^†^	-0.31	0.03^†^	0.73	-0.02^†^	0.46	0.18^†^
ankle velocity (deg/s)	0.45^†^	0.10^†^	0.03^†^	-0.03^†^	-0.07^†^	-0.14^†^	0.35	-0.04^†^	-0.23	0.01^†^
stride time (s)	0.35	0.23	0.42	-0.02^†^	0.31	-0.08^†^	-0.73	0.08^†^	-0.46	-0.15^†^
stride length (m)	-0.27	0.14^†^	0.42	-0.06^†^	0.18^†^	-0.04^†^	0.74	-0.01^†^	-0.30	-0.14^†^

^†^ denotes non-significant correlations

The dispersion of data is captured by the coefficient of variation in Table 5. Normalization succeeded in significantly reducing the dispersion of data for all gait and mobility measures. The ability of the multiple linear regression models to accurately predict gait parameters and mobility measures clearly reflects the normalization’s capacity to reduce data dispersion. Accurate models (in terms of adjusted *R*^2^), such as those for cadence, stride time or 10mWT were able to reduce data dispersion by 50% to 69%. Similarly, the model for stride length and TUG tests reduced data dispersion by 42% and 33%, respectively. Less predictive models, such as those for stand-to-sit, sit-to-stand and ankle velocity, reduced data dispersion by 4% to 15%.

**Table 5:** Coefficient of variation indicating the dispersion of data before and after normalization.

Coefficient of Variation (%)	Raw Data			Normalized Data			Significance
	Mean	95% CI	SE	Mean	95% CI	SE	
***Mobility Measures***							
3mTUG (s)	17.16	[15.46, 18.81]	0.864	12.37	[13.53, 16.59]	0.734	< 0.001
7mTUG (s)	15.09	[13.53, 16.59]	0.736	9.96	[8.89, 10.93]	0.510	< 0.001
10mWT (s)	15.72	[13.78, 17.51]	0.931	4.85	[3.92, 5.61]	0.433	< 0.001
sit-to-stand (s)	27.98	[25.11, 30.79]	1.476	23.71	[21.42, 25.96]	1.157	< 0.001
stand-to-sit (s)	25.04	[22.79, 27.368]	1.206	23.98	[21.40, 26.53]	1.303	< 0.001
***Gait Parameters***							
cadence (steps/min)	8.94	[7.60, 10.27]	0.663	4.39	[3.79, 4.96]	0.299	< 0.001
ankle velocity (deg/s)	9.64	[8.45, 10.71]	0.56	8.12	[7.25, 8.94]	0.439	< 0.001
stride time (s)	9.43	[7.59, 11.19]	0.953	4.63	[3.92, 5.26]	0.354	< 0.001
stride length (m)	10.25	[8.80, 11.67]	0.751	5.89	[4.92, 6.81]	0.493	< 0.001

## Discussion

In order to account for anthropometric differences among subjects within or between cohorts, this study attempted to de-correlate gait parameters and mobility measures from such influences using a normalization procedure that relies on multiple linear regression models. The derived models indicate the significant effects of various anthropometric measurements on performance. However, self-selected walking speeds had a profound role in predicting gait parameters and mobility measures as well. After all, a comfortable walking pace is subject to an individual’s interpretation, and walking speed has been shown to impact spatio-temporal gait parameters [22, 23]. The fact that walking speed remained significant for the stand-to-sit and sit-to-stand motions where no walking is performed suggests that it describes more than merely a speed component. Higher motivational levels and superior gait capabilities in form of a reliable sense of balance and coordination ought to have an effect on a subject’s self-selected walking speed. Hence, a highly motivated subject whose comfortable pace is faster than average will most likely stand up in a faster motion as well. Overall, walking speed established itself as the most prominent effect within the resulting linear regression models, which suggests that it should be accounted for in some way when evaluating the performance on gait parameters and mobility measures.

With regard to the resulting linear regression models for mobility measures, both TUG variants, as well as the stand-to-sit and sit-to-stand motions, remained significantly influenced by a subject’s age even after accounting for a subject’s self-selected walking speed. The 3mTUG and 7mTUG performances were slower by 0.29 s and 0.39 s, respectively, for every 10 years increase in age. The TUGs dependence on age is well known, and the resulting linear regression models predict values within previously published normative ranges [2, 17, 31]. However, the effect of age on the TUG performance is most likely larger than what our regression models suggest when applied to elderly subjects. The aforementioned studies on normative values for TUG tests are derived from elderly cohorts and indicate an approximate slowdown of 1 s for every 10 years increase in age. The derived regression models on the other hand attribute part of the decreasing performance to a reduced self-selected walking speed. Within elderly cohorts, the highly progressed age inevitably results in a reduced gait capacity which affects a subject’s self-selected walking speed. Such a correlation between age and walking speed was observed within our cohort (*r*_*s*_ = -0.38, p < 0.05), and is likely to be even larger in cohorts comprising elderly individuals only. Hence, if we were to remove walking speed from our linear regression model, the age’s impact on the TUG performance should increase. The linear regression models without walking speed in Table 3 indicate exactly that. In these models, the impact of age on the 3mTUG and 7mTUG performances increases to 0.49 s and 0.70 s for every 10 years in age, respectively.

The resulting linear regression models for gait parameters on the other hand indicated no significant dependence on a subject’s age. It was height in conjunction with walking speed that determined all gait parameters with highly accurate models except for ankle velocity. Every 10 cm increase in height roughly extended the stride length by 5 cm and reduced cadence by 5.6 steps/min. Nonetheless, previous studies have indicated a significant correlation of age with gait parameters [15, 32–35]. And although gait parameters were noted to correlate with age (|*r*_*s*_| > 0.27) in this study as well, the correlation with self-selected walking speed dominated (|*r*_*s*_| > 0.35). Having employed the backward elimination method for model selection, age ended up being removed in favor of walking speed. The final regression models explain 66% to 76% in the observed variance using self-selected walking speed. Similarly, Wahid et al.’s regression models for gait parameters reached up to 75% using walking speed as an independent variable [21]. Contrariwise, Samson et al. provides an example for when age instead of walking speed is employed in a linear regression model to predict gait parameters [15]. For cadence and stride length, their models explained a comparably low 30% to 59% of the observed variation.

The noted influences of anthropometric measurements and walking speeds have been successfully de-correlated from gait parameters and mobility measures through normalization. There were no significant correlations after normalization with respect to the mobility measures. For gait parameters, no significant correlations remained except for age in cadence and stride time. The overall result of reducing correlations from |*r*_*s*_| < 0.74 to |*r*_*s*_| < 0.23 and reducing the dispersion of data by up to 69% indicates the favorable outcome of using normalization. The use of the derived regression models hence motivate their employment for normalization of data, allowing for accurate comparisons of gait parameters and mobility measures between cohorts of varying anthropometric measurements.

There are several limitations to the study that need to be addressed in order to adequately interpret our findings. First, the sit-to-stand, stand-to-sit and ankle velocity linear regression models are comparably imprecise in their ability to predict a subject’s outcome given the anthropometric measurements age, height, body mass and gender, along with the self-selected walking speed. A possibility to augment the ankle velocity model is to include leg length, rather than height, as an independent variable [19]. Second, there are other factors than anthropometric measurements and self-selected walking speed that play a role in predicitng gait ability. Examples include muscle strength and cognition [3, 36]. Third, the derived multiple linear regression models are incapable of capturing non-linear effects. Non-linearity has been observed in gait variability of an elderly cohort [32], and any present non-linearity within regular gait cannot be captured by linear models. Fourth, when comparing our results to those of other studies, differences in measurement, laboratory or trial related factors between our study and others might be responsible for some of the data variance observed. Finally, the sample size of 87 subjects may limit the ability to obtain highly accurate regression models. Nonetheless, the derived regression models’ ability to accurately predict its outcomes are comparable to previously published regression models [15, 21].

## Conclusion

Differences in anthropometric measurements impede accurate physiotherapeutic assessment and gait characterization. Mobility measures are predominantly affected by age and walking speed, while gait parameters are determined by height and walking speed. Through normalization, gait parameters and mobility measures can be de-correlated from anthropometric measurements and self-selected walking speeds. Employing multiple linear regression models for normalization purposes, the normalized gait parameters and mobility measures indicate a reduction in data dispersion and removal of significant correlations with anthropometric measurements. Hence, the resulting normalized measures augment the capability to compare subjects with different anthropometric measurements.
